# Supplementary material for: Pregnancy Outcomes and SARS-CoV-2 Infection: The Spanish Obstetric Emergency Group Study
Source: Viruses. 2021 May 7;13(5):853. doi: 10.3390/v13050853 (PMC8151603; doi:10.3390/v13050853)
Supplement: Supplementary file 1 [file viruses-13-00853-s001.zip › viruses-1187358-supplementary.pdf]

Supplementary Table S1. List of hospitals members of the Spanish Obstetric Emergency Group included in this study (n = 78).

HOSPITAL
AGSE Hospital Axarquia
Complejo Asistencial de León
Complejo Hospitalario A Coruña
Complejo Hospitalario de Jaén
Complejo Hospitalario San Millán y San Pedro
Complejo Hospitalario Universitario de Pontevedra
Complexo Hospitalario Universitario de Ourense
HM Hospital Nuevo Belén
Hospital Alto Guadalquivir
Hospital Álvaro Cunqueiro (CHUVI)
Hospital Arnau de Vilanova
Hospital Clínico de Santiago de Compostela
Hospital Clínico San Carlos
Hospital Clínico San Cecilio (Complejo Hospitalario Universitario de Granada)
Hospital Clínico Universitario de Valladolid
Hospital Clínico Universitario Virgen de la Arrixaca
Hospital Costa del Sol
Hospital d'Inca
Hospital de la Santa Creu i Sant Pau
Hospital de Poniente
Hospital de Santa Caterina
Hospital de Son Llàtzer
Hospital de Torrejón
Hospital de Vinalopó
Hospital del Mar
Hospital del Tajo
Hospital do Barbanza
Hospital Doce de Octubre
Hospital Donostia
Hospital General de L'Hospitalet
Hospital General La Mancha Centro
Hospital General Universitario de Ciudad Real
Hospital General Universitario Gregorio Marañón
Hospital General Universitario Santa Lucía
Hospital Infanta Margarita
Hospital Jerez de la Frontera
Hospital La Fe
Hospital La Línea
Hospital Parc Taulí
Hospital Puerta de Hierro
Hospital Quirón Pozuelo de Alarcón
Hospital Quirónsalud Málaga
Hospital Rafael Méndez
Hospital Regional Universitario de Málaga
Hospital Reina Sofía
Hospital San Pedro Alcántara

Hospital Sant Joan de Reus
Hospital Santa Ana
Hospital Universitari Dexeus - Grupo Quirónsalud
Hospital Universitari Germans Trias i Pujol
Hospital Universitario Araba-Txagorritxu
Hospital Universitario Central de Asturias
Hospital Universitario de Basurto
Hospital Universitario de Burgos
Hospital Universitario de Cabueñes
Hospital Universitario de Ceuta
Hospital Universitario de Ferrol
Hospital Universitario de Fuenlabrada
Hospital Universitario de Girona Doctor Josep Trueta
Hospital Universitario de Salamanca
Hospital Universitario de Tarragona Juan XXIII
Hospital Universitario de Torrevieja
Hospital Universitario Doctor Peset
Hospital Universitario Infanta Sofía
Hospital Universitario La Paz
Hospital Universitario Puerta del Mar
Hospital Universitario Río Hortega
Hospital Universitario Son Espases
Hospital Universitario Torrecárdenas
Hospital Universitario Virgen de las Nieves
Hospital Universitario Virgen de Valme
Hospital Universitario Virgen del Rocio
Hospital Universitario Virgen Macarena
Hospital Univesitario de Getafe
Hospital Univesitario Severo Ochoa
Hospital Viamed Santa Ángela de la Cruz
Hospital Virgen de la Concha
Hospital Virgen de la Luz

Supplementary Table S2. STROBE Statement—checklist of items that should be included in reports of observational studies

	Item No.	Recommendation	Page No.	Relevant text from manuscript
**Title and abstract**	1	(*a*) Indicate the study's design with a commonly used term in the title or the abstract	1	
		(*b*) Provide in the abstract an informative and balanced summary of what was done and what was found	1	
		**Introduction**		
Background/rationale	2	Explain the scientific background and rationale for the investigation being reported	2	
Objectives	3	State specific objectives, including any prespecified hypotheses	2	
		**Methods**		
Study design	4	Present key elements of study design early in the paper	2-3	
Setting	5	Describe the setting, locations, and relevant dates, including periods of recruitment, exposure, follow-up, and data collection	2-3, Figure 1 and Supplementary Table S1	
Participants	6	(*a*) *Cohort study*—Give the eligibility criteria, and the sources and methods of selection of participants. Describe methods of follow-up<br>*Case-control study*—Give the eligibility criteria, and the sources and methods of case ascertainment and control selection. Give the rationale for the choice of cases and controls<br>*Cross-sectional study*—Give the eligibility criteria, and the sources and methods of selection of participants	2-3 and Figure 1	
		(*b*) *Cohort study*—For matched studies, give matching criteria and number of exposed and unexposed<br>*Case-control study*—For matched studies, give matching criteria and the number of controls per case		
Variables	7	Clearly define all outcomes, exposures, predictors, potential confounders, and effect modifiers. Give diagnostic criteria, if applicable	2-3 and registry protocol: https://osf.io/xspwq/ https://osf.io/m5yps/	
Data sources/ measurement	8*	For each variable of interest, give sources of data and details of methods of assessment (measurement). Describe comparability of assessment methods if there is more than one group	2-3 and registry protocol: https://osf.io/xspwq/ https://osf.io/m5yps/	
Bias	9	Describe any efforts to address potential sources of bias	2-3 and 9	
Study size	10	Explain how the study size was arrived at	2-3 and Figure 1	

Quantitative variables	11	Explain how quantitative variables were handled in the analyses. If applicable, describe which groupings were chosen and why	2-3, Tables 1-2 and registry protocol: https://osf.io/xspwq/ https://osf.io/m5yps/
Statistical methods	12	(*a*) Describe all statistical methods, including those used to control for confounding	2-3 and registry protocol: https://osf.io/xspwq/ https://osf.io/m5yps/
		(*b*) Describe any methods used to examine subgroups and interactions	2-3
		(*c*) Explain how missing data were addressed	2-3 and Tables 1-2
		(*d*) *Cohort study*—If applicable, explain how loss to follow-up was addressed *Case-control study*—If applicable, explain how matching of cases and controls was addressed *Cross-sectional study*—If applicable, describe analytical methods taking account of sampling strategy	
		(*e*) Describe any sensitivity analyses	No sensitivity analysis was carried out
		**Results**	
Participants	13*	(a) Report numbers of individuals at each stage of study—eg numbers potentially eligible, examined for eligibility, confirmed eligible, included in the study, completing follow-up, and analysed	4 and Figure 1
		(b) Give reasons for non-participation at each stage	Figure 1
		(c) Consider use of a flow diagram	Figure 1
Descriptive data	14*	(a) Give characteristics of study participants (eg demographic, clinical, social) and information on exposures and potential confounders	4, Figure 1 and Table 1
		(b) Indicate number of participants with missing data for each variable of interest	Tables 1-2
		(c) *Cohort study*—Summarise follow-up time (eg, average and total amount)	2-3
Outcome data	15*	*Cohort study*—Report numbers of outcome events or summary measures over time	5-7 and Table 2
		Case-control study—Report numbers in each exposure category, or summary measures of exposure	
		Cross-sectional study—Report numbers of outcome events or summary measures	
Main results	16	(*a*) Give unadjusted estimates and, if applicable, confounder-adjusted estimates and their precision (eg, 95% confidence interval). Make clear which confounders were adjusted for and why they were included	5-7 and Table 2
		(*b*) Report category boundaries when continuous variables were categorized	Tables 1-2

		(*c*) If relevant, consider translating estimates of relative risk into absolute risk for a meaningful time period	
Other analyses	17	Report other analyses done—eg analyses of subgroups and interactions, and sensitivity analyses	7
**Discussion**			
Key results	18	Summarise key results with reference to study objectives	7-8
Limitations	19	Discuss limitations of the study, taking into account sources of potential bias or imprecision. Discuss both direction and magnitude of any potential bias	8-9
Interpretation	20	Give a cautious overall interpretation of results considering objectives, limitations, multiplicity of analyses, results from similar studies, and other relevant evidence	7-9
Generalisability	21	Discuss the generalisability (external validity) of the study results	9
**Other information**			
Funding	22	Give the source of funding and the role of the funders for the present study and, if applicable, for the original study on which the present article is based	9

*Give information separately for cases and controls in case-control studies and, if applicable, for exposed and unexposed groups in cohort and cross-sectional studies.

Note: An Explanation and Elaboration article discusses each checklist item and gives methodological background and published examples of transparent reporting. The STROBE checklist is best used in conjunction with this article (freely available on the Web sites of PLoS Medicine at http://www.plosmedicine.org/, Annals of Internal Medicine at http://www.annals.org/, and Epidemiology at http://www.epidem.com/). Information on the STROBE Initiative is available at www.strobe-statement.org.
